# A rare cause of narrow QRS complex tachycardia: the tortoise and the hare

**DOI:** 10.1007/s12471-022-01685-9

**Published:** 2022-04-29

**Authors:** S. C. Yap

**Affiliations:** Department of Cardiology, Erasmus Medical Centre, University Medical Centre Rotterdam, Rotterdam, The Netherlands

A 39-year-old woman presented to our emergency department with palpitations. She is known with premature atrial beats and a normal transthoracic echocardiogram. During the past year she developed palpitations, in particular following her pregnancy. Currently, she is not using any heart medication. Her family history was unremarkable. Her vital signs and physical exam were normal. Her initial ECG at the emergency department demonstrated a narrow QRS complex tachycardia of 186 beats per minute (Fig. 1). After 50 mg metoprolol, her heart returned to normal sinus rhythm (Fig. 2).

**Fig. 1:**
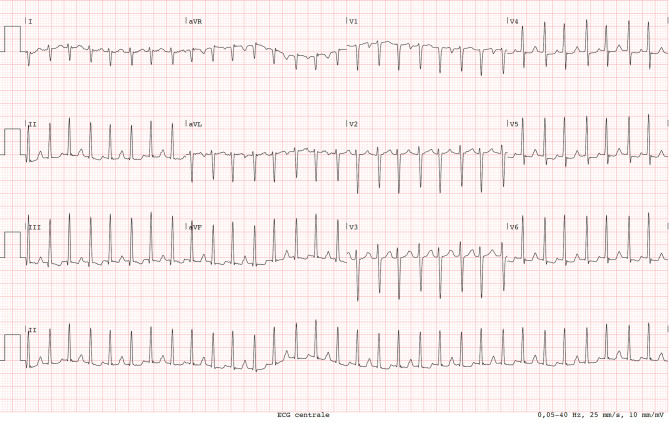
Initial 12-lead ECG at the emergency department demonstrating a regular narrow QRS tachycardia

**Fig. 2:**
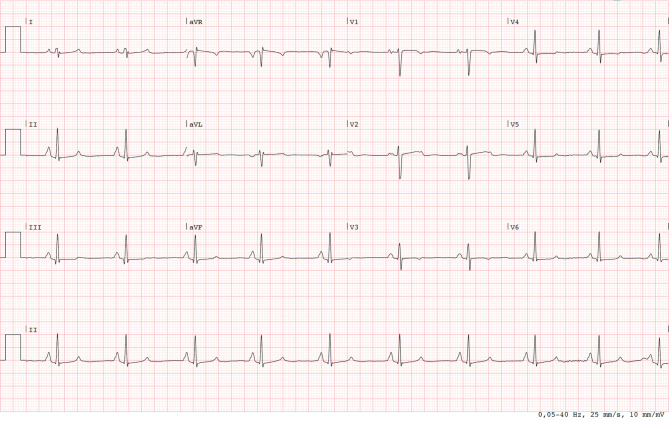
The 12-lead ECG after metoprolol

## Question

What is the mechanism of the narrow QRS complex tachycardia?

## Answer

You will find the answer elsewhere in this issue.

